# Linking Annual N_2_O Emission in Organic Soils to Mineral Nitrogen Input as Estimated by Heterotrophic Respiration and Soil C/N Ratio

**DOI:** 10.1371/journal.pone.0096572

**Published:** 2014-05-05

**Authors:** Zhijian Mu, Aiying Huang, Jiupai Ni, Deti Xie

**Affiliations:** 1 Chongqing Key Laboratory of Soil Multi-scale Interfacial Processes, College of Resources & Environment, Southwest University, Chongqing, China; 2 College of Agronomy & Biotechnology, Southwest University, Chongqing, China; 3 Chongqing Engineering Research Center for Agricultural Non-point Source Pollution Control in Three -Gorges Region, College of Resources & Environment, Southwest University, Chongqing, China; North Carolina State University, United States of America

## Abstract

Organic soils are an important source of N_2_O, but global estimates of these fluxes remain uncertain because measurements are sparse. We tested the hypothesis that N_2_O fluxes can be predicted from estimates of mineral nitrogen input, calculated from readily-available measurements of CO_2_ flux and soil C/N ratio. From studies of organic soils throughout the world, we compiled a data set of annual CO_2_ and N_2_O fluxes which were measured concurrently. The input of soil mineral nitrogen in these studies was estimated from applied fertilizer nitrogen and organic nitrogen mineralization. The latter was calculated by dividing the rate of soil heterotrophic respiration by soil C/N ratio. This index of mineral nitrogen input explained up to 69% of the overall variability of N_2_O fluxes, whereas CO_2_ flux or soil C/N ratio alone explained only 49% and 36% of the variability, respectively. Including water table level in the model, along with mineral nitrogen input, further improved the model with the explanatory proportion of variability in N_2_O flux increasing to 75%. Unlike grassland or cropland soils, forest soils were evidently nitrogen-limited, so water table level had no significant effect on N_2_O flux. Our proposed approach, which uses the product of soil-derived CO_2_ flux and the inverse of soil C/N ratio as a proxy for nitrogen mineralization, shows promise for estimating regional or global N_2_O fluxes from organic soils, although some further enhancements may be warranted.

## Introduction

Although organic soils occupy only 3% of the Earth's land area, they contain approximately 40% (610 Pg) of the terrestrial soil organic carbon (SOC) [1]. Climate warming and human disturbance such as drainage and cultivation are expected to accelerate carbon decomposition in organic soils, and the decomposition of SOC can facilitate the release of mineral nitrogen which can then be utilized by denitrifying and nitrifying bacteria to produce the potent greenhouse gas N_2_O [2], [3]. N_2_O emissions from organic soils under agricultural use in Nordic countries were on average four times higher than those from mineral soils, indicating that N_2_O derived from SOC decomposition dominates overall fluxes [4]. However, no consistent and quantitative relationship has been reported for N_2_O emission and organic carbon decomposition in organic soils.

Organic carbon and nitrogen in soils, plant and microbial biomass are usually covalently bonded at relatively constant ratios. It is thus logical to expect that N_2_O and CO_2_ originated from SOC decomposition should be closely linked. Some studies have indeed found a significant relationship between soil N_2_O and CO_2_ emissions at the site level [5], [6]. This relationship, however, was weaker when data were pooled across sites or ecosystems[7], [8]. The variability of soil C/N ratio may be one of the important factors undermining the correlation for organic soils. The C/N ratio in organic soils ranges from 50∼100 in weakly decomposed peat to 12∼35 in highly decomposed peat [9]. The supply of mineral nitrogen from SOC decomposition is the outcome of two concurrent and oppositely directed microbial processes – nitrogen mineralization and immobilization [10]. Soils with a high C/N ratio may be characterized by rapid immobilization of nitrogen and soils with a low C/N ratio by higher net nitrogen mineralization and a surplus of available NH_4_^+^ and NO_3_^−^ [11]. A negative relationship has accordingly been shown for C/N ratio of soils and N_2_O fluxes [9]. Similar to the relationship between N_2_O and CO_2_ emissions, the correlation of N_2_O emission with soil C/N ratio tended to be weak when the data from different sites at larger scales were included [4], [12], which makes it difficult to scale up N_2_O fluxes by CO_2_ emissions or C/N ratio alone from individual sites to regional scales. In view of the coupling of soil carbon and nitrogen processes and the bridging function of C/N ratio, we hypothesized that a combination of soil CO_2_ emission and C/N ratio would likely provide better measurements of N_2_O emission at larger scales. In fact, Mu et al. [13] have linked N_2_O flux to soil mineral nitrogen as estimated by CO_2_ emission and C/N ratio for agricultural mineral soils. To our knowledge, no such kind of attempt has ever been made for organic soils. The aim of this study was therefore to determine: 1) if N_2_O flux from organic soils is related to soil mineral nitrogen input estimated from heterotrophic respiration divided by soil C/N ratio (a derived measure of soil nitrogen mineralization) plus fertilizer nitrogen; and 2) whether or not the relationship is sufficiently robust to serve as an approach for estimating N_2_O flux from organic soils.

## Materials and Methods

### Data source

To test the hypothesis, we collected journal-published data of N_2_O and CO_2_ emissions measured simultaneously in the fields on peatlands or histosols for which the carbon and nitrogen content or ratio of the organic matter in the upper layers of the soil has been reported. Occasional and short-period flux measurements were not used and only data on annual emissions were considered. For long-term measurements, we used annual estimates rather than multi-year averages to reflect temporal variability. Annual emissions were directly reported by authors or estimated from points in the figures of publications. The final dataset comprised of 122 field measurements from 28 geographical sites (Table S1). Of all data, only 12 measurements at 9 sites were from the tropical regions and the rest were from the temperate regions. Most of the flux measurements were made using closed chamber technique with sampling frequency varying from 1–3 times per week to once per month. Other factors such as soil pH and water table level, if reported, were also recorded in the database. Readers should refer to the original papers for a more complete presentation of the data.

### Estimation of soil mineral nitrogen input

The CO_2_ emission measured in bare soils can be taken as the proxy of SOC decomposition or heterotrophic respiration [14]. There are limited studies in which CO_2_ emission was measured in bare soils (Table S1). For the CO_2_ emissions measured in soils with plants, the contribution of heterotrophic respiration or SOC decomposition was estimated using the following equation adapted from Bond-Lamberty and Thomson [15]:
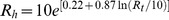
(1)where R_h_ is heterotrophic respiration and R_t_ is total soil respiration (kg C ha^−1^ yr^−1^).

The nitrogen mineralization rate from soil organic matter was then calculated using the following equation [13]:
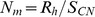
(2)where N_m_ is the gross nitrogen mineralization (kg N ha^−1^ yr^−1^) and S_CN_ is soil C/N ratio.

The mineralized nitrogen from soil organic matter decomposition and the inorganic nitrogen from chemical fertilizers constitute the total input of soil mineral nitrogen (N_mf_). Atmospheric nitrogen deposition, as another important external source of soil mineral nitrogen, was not considered for our study since there were few papers reporting it.

### Statistical analysis

The dataset in the current study is of unbalanced nature with observations collected from peer-reviewed papers rather than from systematically designed experiments. Accordingly, the effects of soil mineral nitrogen input and other variables on N_2_O flux were analyzed using the mixed model-REML estimation method of SAS/MIXED procedure (version 9.3), which is suitable for handling unbalanced data. The values of N_2_O flux were first natural-log transformed to normalize their distribution and then analyzed by the following model: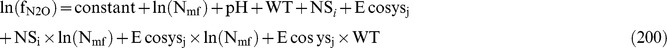 where f_N2O_ is the N_2_O flux; N_mf_, pH, WT, NS_i_ and Ecosys_j_ are the fixed effects of mineral nitrogen input, soil pH, water table level, nitrogen source (i is mineralized nitrogen only or a combination of mineralized nitrogen and inorganic nitrogen from chemical fertilizers), and ecosystem type (j is forest or non-forest type), respectively. A preliminary check of the data showed that the general trend of N_2_O flux in forest system differed from grass and cropland, so the ecosystems were simply classified into two subclasses as forest and non-forest. Some two-factor interactions were also included in the model. A significant level of *p* = 0.05 was used to determine if a given variable or interactive effect was kept in the model to further seek solutions for fixed effects. Four negative values of N_2_O flux reported by Inubushi et al. [16] and Mojeremane et al. [17] can not be subjected to log-transformation and were not included in the analysis. In addition to determination coefficient (i.e., R^2^ value), concordance between observed N_2_O fluxes and model fits was also analyzed using Lin's concordance correlation coefficient (CCC, Stata SE 12.0) to assess the goodness-of-fit of the finalized models. The resulting CCC was interpreted using the benchmarks described by Klevens et al. [18] as follows: <0.20 is considered virtually no agreement; 0.21–0.40 is considered slight; 0.41–0.60 is considered fair; 0.61–0.80 is considered moderate; and 0.81–0.99 is substantial.

## Results

As shown in Table 1, soil pH, soil mineral nitrogen source (NS) and ecosystem type did not affect the annual N_2_O flux (*p*>0.05), while the input of soil mineral nitrogen (N_mf_) and water table level (WT) had significant effects on N_2_O flux (*p*<0.01). The F value of N_mf_ was the biggest, indicating the input of soil mineral nitrogen was the main factor controlling N_2_O emission in organic soils. The two-factor interactive effects between NS, N_mf_, WT and ecosystem type on N_2_O flux were not statistically significant (*p*>0.05).

**Table 1:** Results of type III tests of fixed effects.

Effect	Numerator DF	Denominator DF	F Value	Pr>F
N_mf_	1	96	13.16	0.0005
pH	1	96	1.43	0.2344
WT	1	96	5.15	0.0255
NS	1	96	0.10	0.7472
Ecosystem	1	96	0.70	0.4040
NS×N_mf_	1	96	0.11	0.7426
WT×N_mf_	1	96	3.20	0.0767
Ecosystem×N_mf_	1	96	0.21	0.6506
Ecosystem×WT	1	96	2.17	0.1437

N_mf_, the mineral nitrogen input to soil; WT, water table level; NS, the source of soil mineral nitrogen.

**Figure 1:**
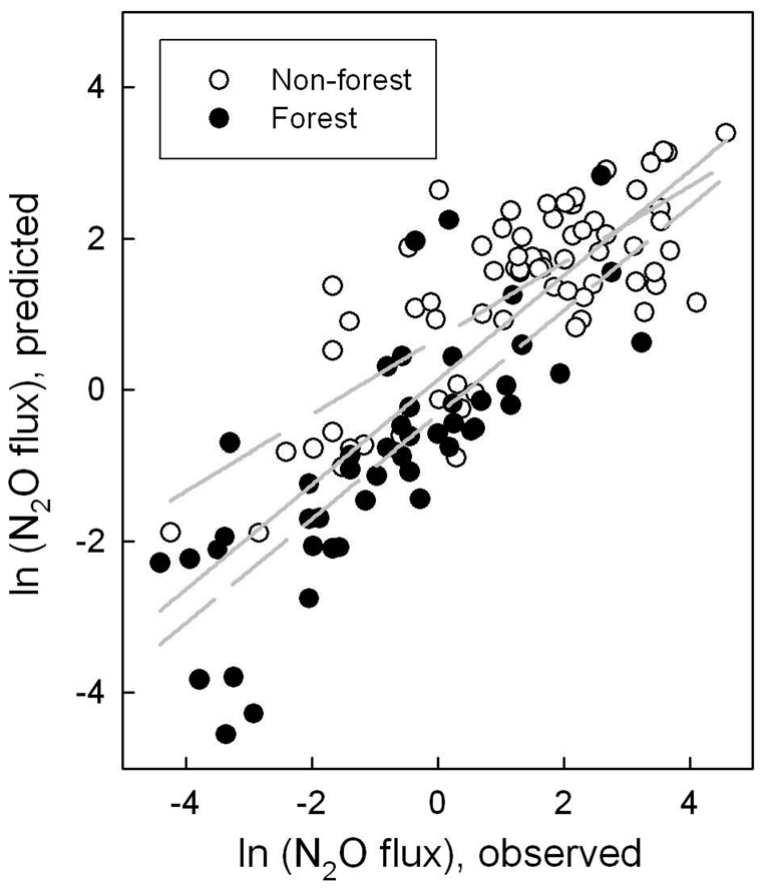
Correlation of observed fluxes of N_2_O from organic soils and predicted values (kg N_2_O-N ha^−1^) by model 1 as presented in Table 2: ln(N_2_O flux) = 1.8685 ln(N_mf_)−9.0314. Solid line shows linear regression fit for the overall data: y = 0.69x+0.13, R^2^ = 0.69. Long-dashed line shows linear regression fit for the non-forest system: y = 0.50x+0.68, R^2^ = 0.59. Short-dashed line shows linear regression fit for the forest system: y = 0.69x−0.33, R^2^ = 0.63.

**Figure 2:**
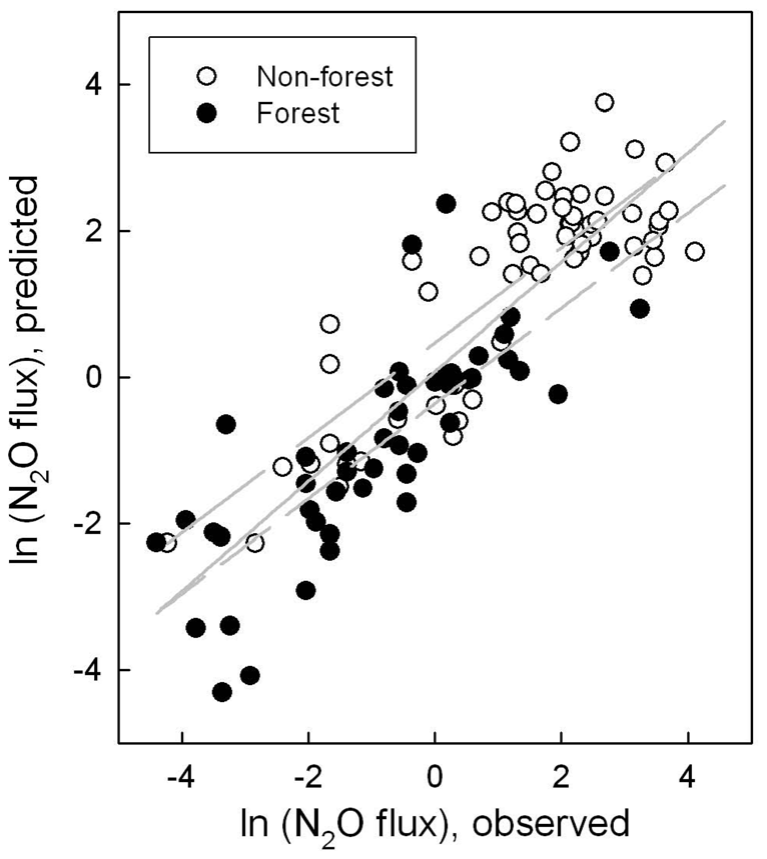
Correlation of observed fluxes of N_2_O from organic soils and predicted values (kg N_2_O-N ha^−1^) by model 2 as presented in Table 2: ln(N_2_O flux) = 1.5374 ln(N_mf_)−0.0221 WT−8.2334. Solid line shows linear regression fit for the overall data: y = 0.75x+0.08, R^2^ = 0.75. Long-dashed line shows linear regression fit for the non-forest system: y = 0.65x+0.48, R^2^ = 0.69. Short-dashed line shows linear regression fit for the forest system: y = 0.65x−0.35, R^2^ = 0.63.

Only the significant variables were then kept in the model to solve the estimates for their effects. Two models with different combinations of independent variables are shown in Table 2. The first model was the simplest one with N_mf_ as the single independent variable. The second model was expanded by adding the effect of water table level. The 95% confidence intervals of the estimated effect of N_mf_ were overlapped for different models. The models indicated that N_2_O flux was positively correlated with N_mf_ and negatively with water table level. Using the estimated effects and the variables in the dataset allowed a comparison between predicted and observed annual N_2_O fluxes from organic soils. The variable N_mf_ explained up to 69% of the variability in the overall data of observed N_2_O fluxes (Fig. 1), while the addition of water table level increased the explanatory ability to 75% (Fig. 2). When the overall data were further divided by ecosystem types, the performance of models was somewhat different (Fig. 1 & 2). For forest, the determination coefficient (R^2^) was nearly stable at the value of 0.63 for both models. In contrast, the introduction of water table level into models slightly improved the fitted results for non-forest systems with R^2^ values increasing from 0.59 to 0.69. This indicated that the input of mineral nitrogen was the most important predictor of N_2_O flux, while water table level was a weak predictor of N_2_O flux and appeared to be dependent on ecosystem type.

**Table 2:** Solutions for fixed effects of the models with log-transformed N_2_O flux as dependent variable.

Model	Effect	Estimate	SE	DF	t Value	Pr>|t|	95% confidence	
							Lower	Upper
1	Intercept	−9.0314	0.5952	116	−15.17	<.0001	−10.2102	−7.8526
	ln (N_mf_)	1.8685	0.1157	116	16.15	<.0001	1.6393	2.0976
								
2	Intercept	−8.2334	0.6222	103	−13.23	<.0001	−9.4675	−6.9994
	ln (N_mf_)	1.5374	0.1479	103	10.39	<.0001	1.2441	1.8308
	WT	−0.0221	0.0053	103	−4.14	<.0001	−0.0326	−0.0115

N_mf_, the mineral nitrogen input to soil (kg N ha^−1^ yr^−1^); WT, water table level (cm).

The slope of regression lines in Fig. 1 & 2 ranged from 0.50 to 0.75, indicating that the relationship strays from the ideal 1∶1 line. Therefore the concordance correlation coefficient (CCC) between observed and predicted N_2_O fluxes was calculated to measure robustness of the models. For the overall data with log-transformation, the concordance was substantial with the CCC ranging from 0.82 to 0.86 for the two models. When the log-transformed data were converted to actual N_2_O fluxes, however, the cluster of fluxes greater than 15.0 kg N ha^−1^ yr^−1^ was found to be distinctly underestimated. The CCC for this cluster of data ranged from −0.002 to 0.16 and showed virtually no agreement, suggesting that some important factors responsible for these high fluxes were not accounted for by the models. For the rest of the data (103 fluxes out of 118), the CCC (ranging from 0.63 to 0.68) still showed a moderate concordance.

The variable N_mf_ in the models can be decomposed into soil heterotrophic respiration (R_h_), C/N ratio and inorganic nitrogen rate from chemical fertilizer (N_f_). The mixed procedure analysis indicated that each of these components of N_mf_ had a significant influence on N_2_O flux (*p*<0.001), with R_h_ and N_f_ being positively related to N_2_O flux and C/N ratio negatively related to N_2_O flux. Soil carbon and nitrogen contents, which could replace the variable of C/N ratio, were also significantly negatively or positively correlated with N_2_O flux (*p*<0.001). The fitting efficiency between observed and predicted N_2_O fluxes by models using the above-mentioned components of N_mf_ as inputs were nearly the same as those of models using N_mf_ itself (data not shown).

## Discussion

**Figure 3:**
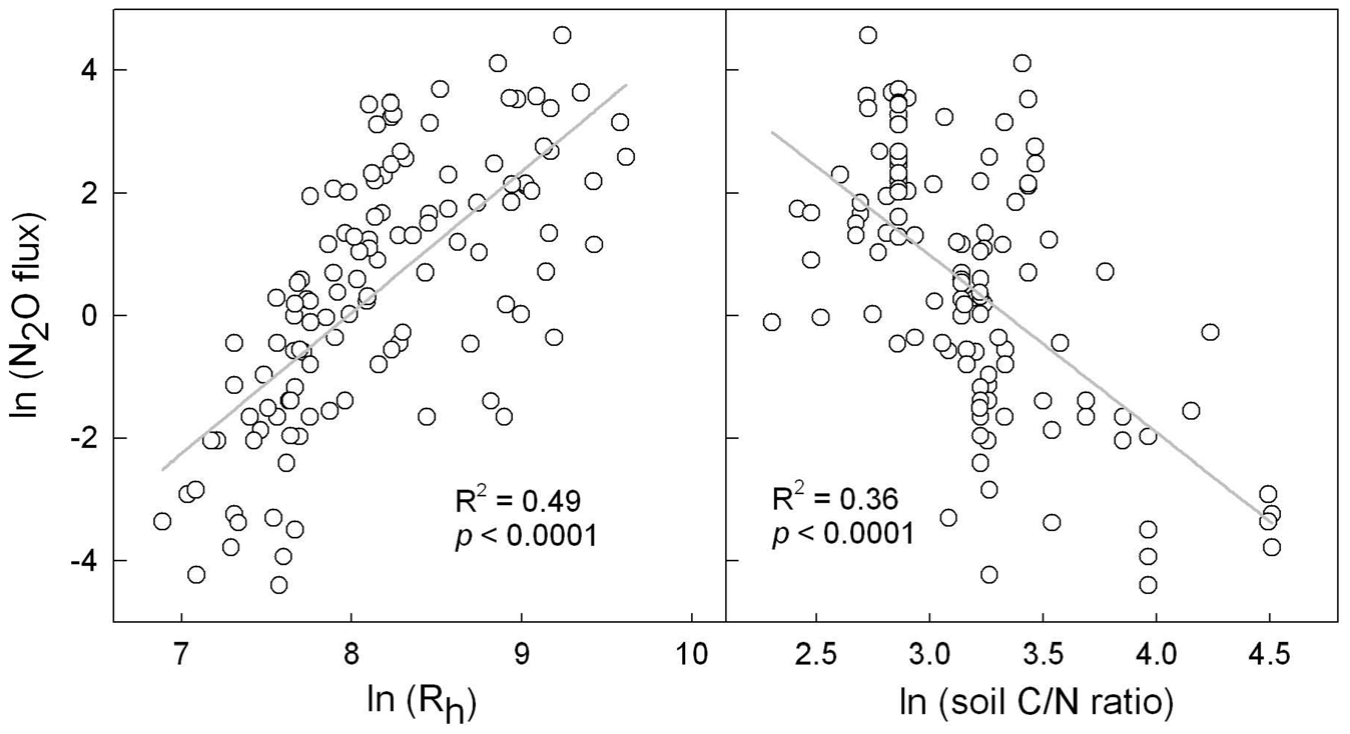
Correlation of observed fluxes of N_2_O with estimated soil heterotrophic respiration (R_h_, left panel) and C/N ratio (right panel).

Previous studies have linked N_2_O flux directly to either CO_2_ flux or soil C/N ratio [5], [8], [9]. In this study, soil CO_2_ emission and C/N ratio were combined to estimate mineral nitrogen input, and the latter accounted for up to 69% of the variability of N_2_O fluxes from organic soils with various properties, land management practices and climates. Soil CO_2_ flux or C/N ratio alone explained only 49% and 36% of the overall variability of N_2_O fluxes, respectively (Fig. 3). This suggests the necessity of combining soil CO_2_ flux and C/N ratio for predicting N_2_O flux on a large scale. Of course, soil CO_2_ flux and C/N ratio can be independently incorporated into the same models, but the interpretation of such models would be relatively complicated and evasive since there are various mechanisms which may explain the control of CO_2_ flux and C/N ratio over N_2_O flux [8], [9], [19]. In contrast, the quotient of soil CO_2_ flux and C/N ratio can well represent in theory the gross nitrogen mineralization [20], and the implication of models using such a quotient as input is straightforward and self-evident in the importance of mineral nitrogen input for regulating soil N_2_O flux. There is no significant difference in the influence of different sources of mineral nitrogen on N_2_O flux (Table 1), suggesting that the simplified models might also be suitable for evaluating the effect of mineral nitrogen from other sources such as atmospheric deposition, though this idea needs further verification.

A negative relationship between N_2_O flux and groundwater level has been observed for individual sites [21], [22], and still holds at a large scale as shown in this study. This is logical simply because high moisture with increasing water table level can limit N_2_O emission from soils due to the low availability of nitrate and/or efficient reduction of N_2_O to N_2_ through denitrification [16], [23], while the lowering of water table increases oxygen penetration into the peat and enhances the decomposition of organic matter, as indicated by the negative relationship between heterotrophic respiration and water table level (R^2^ = 0.31, *p*<0.0001). It has been reported that the control of soil water content or water table level over N_2_O flux is important only when soil is not nitrogen limiting [24], [25]. In this study, the percentage of observations with N_mf_ greater than 150 kg N ha^−1^ was only 19% for forest, but up to 87% for non-forest systems (Table S1). This suggests that forest soil is nitrogen limiting when compared with non-forest systems, which may be responsible for the insensitivity of N_2_O flux to water table level for forest systems (Fig.1& 2). Besides the input of mineral nitrogen, forest differs from non-forest systems in many other factors, such as vegetation, below-/above-ground biomass, litter fall, soil compaction, and land management practices, all of which can influence N_2_O flux but are not considered here due to limited and unsystematic information in literature sources of the current dataset. To fill the gap, ecosystem type was used as a proxy variable that we tried to incorporate into models; however, statistical analysis showed that its effect was not significant (Table 1).

It should be acknowledged that the models described here were dependent on simplifying assumptions that can introduce error. That is, the gross nitrogen mineralization was estimated from carbon mineralization and soil C/N ratio by assuming that the rate of carbon mineralization is the same as the rate of respiration and the C/N ratio of mineralized organic matter is the same as that of the bulk soil organic matter. In fact, carbon and nitrogen mineralization from soils originates from decomposable fractions of organic matter with different C/N ratios [26]. Most likely, the ratio of carbon evolved/nitrogen mineralized is much wider than the bulk soil carbon to nitrogen ratio [27], [28]. This indicates that gross nitrogen mineralization might be over- or under-estimated if bulk soil C/N ratio was used in equation 2. The respiration process is also not exactly identical to carbon mineralization. The amount of carbon that is ultimately lost through respiration depends on how effectively the decomposer community converts mineralized carbon to biomass [29]. Similarly, the amount of nitrogen that is ultimately available to denitrifier or nitrifier for producing N_2_O depends on how effectively the decomposer community converts mineralized nitrogen to biomass and plants compete with microbes for mineral nitrogen [10], [20]. Empirical relationships have been established between nitrogen and carbon mineralization in studies performed usually under laboratory conditions [30]. Different organic matter fractions or their C/N ratios, and varying microbial use efficiency of carbon and nitrogen have also been proposed to predict nitrogen release [20], [29]. However, these relationships are strongly dependent on the experimental conditions in which they have been established. Moreover, the current dataset is based on the in situ measurements in the field environment and contains only the basic information of respiratory carbon and bulk soil C/N ratio, thus necessitating the above-mentioned assumptions to estimate mineralized nitrogen. Such simplifications and assumptions may bring uncertainties, but it is necessary in some cases to understand the general trends and probabilistic nature of the environment [31].

N_2_O emission from soils is of small magnitude and highly variable in space and time, and is thus very difficult to estimate. The measurement of soil N_2_O flux also requires intricate techniques along with a lot of time and labor. In contrast, soil CO_2_ emission is controlled primarily by soil temperature and moisture, and is relatively easy to measure or predict [32], [33]. In addition, the estimates of soil respiration are currently more widely available than those of soil N_2_O emission. The models developed in this study showed a promising approach to estimating N_2_O emission from organic soils by using soil C/N ratio and CO_2_ emission data derived from measurements or biogeochemical modeling. It should be mentioned, however, that several aspects of the information in the current dataset might impose uncertainties on these models. First, soil heterotrophic respiration was simply estimated from total soil respiration using a universal relationship between them [15], but the relative contribution of organic matter decomposition or heterotrophic respiration would vary over time and depend on root respiration of the growing plants [8]. Second, the majority of the global organic soils are distributed in the boreal and sub-arctic regions and about 10%–15% in the tropical countries [1], [3], but most of the current data came from northern Europe, indicating that the models developed in the present study might be biased to the temperate regions.

## Conclusion

A fairly large number of data were collected to explore the relationship between annual N_2_O emission and multiple variables for organic soil by a mixed-model analysis, and the input of soil mineral nitrogen was found to be the most useful predictor for N_2_O flux. Soil mineral nitrogen was supposed to be composed of organic nitrogen mineralization as estimated by CO_2_ emission and soil C/N ratio, thus providing a possibility for upscaling N_2_O emission from organic soils by use of regional soil databases including information on C/N ratio and carbon storage change or CO_2_ emission data. The approach proposed here may have validity as a whole, but needs further evaluation and advancement before practical application due to uncertainties associated with simplifying assumptions and a regionally unbalanced data source. A better understanding of the processes of carbon and nitrogen mineralization and their stoichiometric relationship as well as additional experimental data from organic soils outside of temperate Europe regions will help to improve the relationship established in this study.

## Supporting Information

Table S1 **Annual emissions of N_2_O and CO_2_ from organic soils and estimates of soil mineral nitrogen input.**
(XLS)

## References

[1] Page SE, Rieley J, Banks CJ (2011) Global and regional importance of the tropical peatland carbon pool. Global Change Biol 17: 798–818.

[2] Goldberg SD, Knorr KH, Blodau C, Lischeid G, Gebauer G (2010) Impact of altering the water table height of an acidic fen on N_2_O and NO fluxes and soil concentrations. Global Change Biol 16: 220–233.

[3] Frolking S, Talbot J, Jones MC, Treat CC, Kauffman JB, et al (2011) Peatlands in the Earth's 21^st^ century climate system. Environ Rev 19: 371–396.

[4] Maljanen M, Sigurdsson BD, Guðmundsson J, Óskarsson H, Huttunen JT, et al (2010) Greenhouse gas balances of managed peatlands in the Nordic countries – present knowledge and gaps. Biogeosciences 7: 2711–2738.

[5] Garcia-Montiel DC, Melillo JM, Steudler PA, Neill C, Feigl BJ, et al. (2002) Relationship between N_2_O and CO_2_ emissions from the Amazon Basin. Geophys Res Lett 29 : Art.No. 1090.

[6] Chatskikh D, Olesen JE (2007) Soil tillage enhanced CO_2_ and N_2_O emissions from loamy sand soil under spring barley. Soil Till Res 97: 5–18.

[7] Keller M, Varner R, Dias JD, Silva H, Crill P, et al. (2005) Soil–atmosphere exchange of nitrous oxide, nitric oxide, methane, and carbon dioxide in logged and undisturbed forest in the Tapajos national forest, Brazil. Earth Interact 9 : Art. No. 23.

[8] Xu XF, Tian HQ, Hui DF (2008) Convergence in the relationship of CO_2_ and N_2_O exchanges between soil and atmosphere within terrestrial ecosystems. Global Change Biol 14: 1651–1660.

[9] Klemedtsson L, Von Arnold K, Weslien P, Gundeersen P (2005) Soil CN ratio as a scalar parameter to predict nitrous oxide emissions. Global Change Biol 11: 1142–1147.

[10] Luxhøi J, Bruun S, Stenberg B, Breland TA, Jensen LS (2006) Prediction of gross and net nitrogen mineralization-immobilization-turnover from respiration. Soil Sci Soc Am J 70: 1121–1128.

[11] Bengtsson G, Bengtson P, Månsson KF (2003) Gross nitrogen mineralization-, immobilization-, and nitrification rates as a function of soil C/N ratio and microbial activity. Soil Biol Biochem 35: 143–154.

[12] Ojanen H, Minkkinen K, Alm J, Penttila T (2010) Soil – atmosphere CO_2,_ CH_4_ and N_2_O fluxes in boreal forestry-drained peatlands. Forest Ecol Manage 260: 411–421.

[13] Mu ZJ, Huang AY, Kimura SD, Jin T, Wei SQ, et al (2009) Linking N_2_O emission to soil mineral N as estimated by CO_2_ emission and soil C/N ratio. Soil Biol Biochem 41: 2593–2597.

[14] Hanson PJ, Edwards NT, Garten CT, Andrews JA (2000) Separating root and soil microbial contributions to soil respiration: A review of methods and observations. Biogeochemistry 48: 115–146.

[15] Bond-Lamberty B, Thomson A (2010) A global database of soil respiration data. Biogeosciences 7: 1915–1926.

[16] Inubushi K, Furukawa Y, Hadi A, Purnomo E, Tsuruta H (2003) Seasonal changes of CO_2_, CH_4_ and N_2_O fluxes in relation to land-use change in tropical peatlands located in coastal area of South Kalimantan. Chemosphere 52: 603–608. 12738298 10.1016/S0045-6535(03)00242-X

[17] Mojeremane W, Rees RM, Mencuccini M (2012) The effects of site preparation practices on carbon dioxide, methane and nitrous oxide fluxes from a peaty gley soil. Forestry 85: 1–15.

[18] Klevens J, Trick WE, Kee R, Angulo F, Garcia D, et al (2011) Concordance in the measurement of quality of life and health indicators between two methods of computer-assisted interviews: self-administered and by telephone. Qual Life Res 20: 1179–1186. 21318647 10.1007/s11136-011-9862-2

[19] Rochette P, Tremblay N, Fallon E, Angers DA, Chantigny MH, et al (2010) N_2_O emissions from an irrigated and non-irrigated organic soil in eastern Canada as influenced by N fertilizer addition. Eur J Soil Sci 61: 186–196.

[20] Murphy DV, Recous S, Stockdale EA, Fillery IRP, Jensen LS, et al (2003) Gross nitrogen fluxes in soil: Theory, measurement and application of ^15^N pool dilution techniques. Adv Agron 79: 69–118.

[21] Regina K, Silvola J, Martikainen PJ (1999) Short-term effects of changing water table on N_2_O fluxes from peat monoliths from natural and drained boreal peatlands. Global Change Biol 5: 183–189.

[22] Danevcic T, Mandic-Mulec I, Stres B, Stopar D, Hacin J (2010) Emissions of CO_2_, CH_4_ and N_2_O from southern European peatlands. Soil Biol Biochem 42: 1437–1446.

[23] Maljanen M, Shurpali N, Hytönen J, Mäkiranta P, Aro L, et al (2012) Afforestation does not necessarily reduce nitrous oxide emissions from managed boreal peat soils. Biogeochemistry 108: 199–218.

[24] Smith KA, Thomson PE, Clayton PE, McTaggart IP, Conen F (1998) Effects of temperature, water content and nitrogen fertilization on emissions of nitrous oxide by soil. Atmos Environ 32: 3301–3309.

[25] Weslien P, Klemedtsson AK, Borjesson G, Klemedtsson L (2009) Strong pH influence on N_2_O and CH_4_ fluxes from forested organic soils. Eur J Soil Sci 60: 311–320.

[26] Springob G, Kirchmann H (2003) Bulk soil C to N ratio as a simple measure of net N mineralization from stabilized soil organic matter in sandy arable soils. Soil Biol Biochem 35: 629–632.

[27] Sollins P, Spycher G, Glassman CA (1984) Net nitrogen mineralization from light- and heavy-fraction forest soil organic matter. Soil Biol Biochem 16: 31–37.

[28] Kader MA, Sleutel S, Begum SA, D'Haene K, Jegajeevagan K, et al (2010) Soil organic matter fractionation as a tool for predicting nitrogen mineralization in silty arable soils. Soil Use Manage 26: 494–507.

[29] Manzoni S, Taylor P, Richter A, Porporato A, Ågren GI (2012) Environmental and stoichiometric controls on microbial carbon-use efficiency in soils. New Phytol 196: 79–91. 22924405 10.1111/j.1469-8137.2012.04225.x

[30] Nicolardot B, Recous S, Mary B (2001) Simulation of C and N mineralization during crop residue decomposition: a simple dynamic model based on the C:N ratio of the residues. Plant Soil 228: 83–103.

[31] Yan XY, Yagi K, Akiyama H, Akimoto H (2005) Statisical analysis of the major variables controlling methane emission from rice fields. Global Change Biol 11: 1131–1141.

[32] Lloyd J, Taylor JA (1994) On the temperature-dependence of soil respiration. Funct Ecol 8: 315–323.

[33] Raich JW, Potter CS, Bhagawati D (2002) Interannual variability in global soil respiration, 1984-94. Global Change Biol 8: 800–812.

